# 

**Published:** 1858-03

**Authors:** 


### AMERICAN DENTAL CONVENTION.

*Subjects for discussion at the next Annual Meeting.*

"The business committee appointed at the convention at Boston, would respectfully present the following topics for discussion at the next convention, to be held in Cincinnati, commencing the first Tuesday of August, 1858.

1. The best means of *scuring* and preserving good teeth.
2. *Treatment* of Exposed Nerves.
3. Mechanical Dentistry.
4. Filling Teeth.
5. Miscellaneous.

J. Taft, C. W. Spalding, C. A. Harris, E. Townsend, B. Lord, *Committee.*"

### RECEIPTS FOR VOL. X.

| | |
|---|---|
| Dr. J. F. Wilson | $2 50 |
| " J. Wortman | 2 50 |
| " E. Collins | 3 00 |
| " C. N. Woodward | 3 00 |
| " A. J. Reive | 3 00 |
| " N. H. Ambler | 3 00 |
| " J. R. Walker | 3 00 |
| " J. B. Dunlevy | 3 00 |
| " E, D. Fuller | 3 00 |
| " S. W. Hale | 3 68 |
| Dr. E. F. Tibbetts | 3 00 |
| " M. N. Manlove & Bro | 3 00 |
| " J. K. Rickey | 3 00 |
| " R. S. Barber | 3 00 |
| " N. W. Hicks | 3 00 |
| " S. Mapes | 3 00 |
| " J. Lukins | 3 00 |
| " J. T. Reynolds | 2 50 |
| " W. B. Roberts | 3 00 |

### RECEIPTS FOR VOL. XI.

| | |
|---|---|
| Dr. T. B. Welch | $3 00 |
| " J. C. Emery | 3 00 |
| " J. W. Huffman | 2 00 |
| " A. B Cunningham | 2 00 |
| " A. J. Townsend | 2 00 |
| " W. Bradly | 3 00 |
| " J. F. Wilson | 2 50 |
| " J. Wortman | 2 50 |
| " E. Collins | 3 00 |
| " C. W. Ballard | 3 00 |
| " C. N. Woodward | 3 00 |
| " A. J. Reive | 3 00 |
| " J. R. Walker | 8 00 |
| " W. B. Ingersoll | 3 00 |
| " J. B. Dunlevy | 3 00 |
| " A. S. Talbert | 3 00 |
| " E. D. Fuller | 3 00 |
| " J. B. Williams | 3 00 |
| " F. Searle | 5 00 |
| Dr. M. W. Wortman | 2 00 |
| " E. F. Tibbitts | 3 00 |
| " M. N. Manlove & Bro | 3 00 |
| " D. A. F. Hall | 2 00 |
| " D. S. Mapes | 3 00 |
| " J. P. Ulrey | 3 00 |
| " W. R. Webster | 3 00 |
| " J. Lukins | 3 00 |
| " Geo, W. Keely | 3 00 |
| " J. B. Smith | 3 00 |
| " A. G. Stipher | 3 00 |
| " S. L. Bracy | 2 00 |
| " G. B. Lenoir | 2 00 |
| " B. F. Rosson | 3 00 |
| " J. T. Reynolds | 2 50 |
| " W. B. Roberts (vols. 11 and 12) | 6 00 |
| " A. Phillips | 3 00 |
| " S. B. Brown | 3 00 |

### ELEVENTH VOLUME

OF THE

### DENTAL REGISTER OF THE WEST.

EDITED BY

**J. TAFT and G. WATT.**

TERMS: - - - - - - - - $3 PER ANNUM, IN ADVANCE.

**Advertisements Inserted as follows:**

Oue page, per annum,.......................................................$25 00
One-half page, per annum.................................................. 15 00
Quarter page, per annum,.................................................. 10 00

Shorter periods than one year, by special arragement.

☞Contributions to the pages of the *Register* respectfully solicited.

Exchanges and Correspondents will please address "Dental Register," or "Taft & Watt, Cincinnati, Ohio."

